# Anisotropy of Mechanical Properties of *Pinctada margaritifera* Mollusk Shell

**DOI:** 10.3390/nano10040634

**Published:** 2020-03-28

**Authors:** Martyna Strąg, Łukasz Maj, Magdalena Bieda, Paweł Petrzak, Anna Jarzębska, Jürgen Gluch, Emre Topal, Kristina Kutukova, André Clausner, Wieland Heyn, Katarzyna Berent, Kinga Nalepka, Ehrenfried Zschech, Antonio G. Checa, Krzysztof Sztwiertnia

**Affiliations:** 1 Institute of Metallurgy and Materials Science, Polish Academy of Sciences, 30-059 Krakow, Poland; l.maj@imim.pl (Ł.M.); m.bieda@imim.pl (M.B.); p.petrzak@imim.pl (P.P.); a.jarzebska@imim.pl (A.J.); k.sztwiertnia@imim.pl (K.S.); 2 Fraunhofer IKTS, Institute for Ceramic Technologies and Systems, 01109 Dresden, Germany; juergen.gluch@ikts.fraunhofer.de (J.G.); emre.topal@tu-dresden.de (E.T.); kristina.kutukova@ikts.fraunhofer.de (K.K.); andre.clausner@ikts.fraunhofer.de (A.C.); wieland.heyn@ikts.fraunhofer.de (W.H.); ehrenfried.zschech@ikts.fraunhofer.de (E.Z.); 3 Academic Centre for Materials and Nanotechnology, AGH University of Science and Technology, 30-059 Krakow, Poland; kberent@agh.edu.pl; 4 Department of Strength and Fatigue of Materials and Structures, AGH University of Science and Technology, 30-059 Krakow, Poland; knalepka@agh.edu.pl; 5 Department of Stratigraphy and Paleontology, University of Granada, 18071 Granada, Spain; acheca@ugr.es

**Keywords:** mollusk shells, bivalve, calcite, nano-XCT, electron microscopy, mechanical properties

## Abstract

The mechanical properties such as compressive strength and nanohardness were investigated for *Pinctada margaritifera* mollusk shells. The compressive strength was evaluated through a uniaxial static compression test performed along the load directions parallel and perpendicular to the shell axis, respectively, while the hardness and Young modulus were measured using nanoindentation. In order to observe the crack propagation, for the first time for such material, the in-situ X-ray microscopy (nano-XCT) imaging (together with 3D reconstruction based on the acquired images) during the indentation tests was performed. The results were compared with these obtained during the micro-indentation test done with the help of conventional Vickers indenter and subsequent scanning electron microscopy observations. The results revealed that the cracks formed during the indentation start to propagate in the calcite prism until they reach a ductile organic matrix where most of them are stopped. The obtained results confirm a strong anisotropy of both crack propagation and the mechanical strength caused by the formation of the prismatic structure in the outer layer of *P. margaritifera* shell.

## 1. Introduction

Biocomposites constitute a large group of materials in which a soft organic matrix is reinforced by a second, hard phase. The organic part may deform and extend during stretching while the hard, mineral phase is able to withstand high compression loads acting similar to ceramics. Thus, the combination of the ductility provided by the organic part and the strength and stiffness guaranteed by the mineral phase may be very promising in materials design [1,2]. What is more, these hard and soft components seem to be very weak materials independently while together they exhibit great mechanical and useful properties. It was already shown that the introduction of ductile cobalt into a hard Al_2_O_3_-TiC ceramic material significantly increases the fracture toughness and efficiently suppresses the crack propagation [3]. However, nature, through many years of evolution, has created better composites in which functionality is combined with unique mechanical properties. A great example is the mollusk shell. This complex structure enables effective protection of the animal against the predators by dissipating the energy of dynamic or static load throughout the entire volume of the material. In this way, a catastrophic fracture is delayed. The mollusk shell could be recognized as the natural ceramic-based composite material built of hard calcium carbonate (CaCO_3_) in the crystallographic form of either calcite or aragonite (~95 wt.%) separated by thin layers of a soft organic part containing polysaccharides and proteins (~5 wt.%) [4].

The investigations of the mollusk shells revealed a variety of microstructures depending on the species. The crossed-lamellar structure (a complex hierarchical structure comprising an ordered arrangement of thin lamellas composed of laths of nano-sized fibers) located in the outer parts of shells and the inner nacreous aragonite layers have been the most frequently examined. The reasons are brilliant mechanical properties as well as a possibility to reproduce these materials as components in synthetic, ceramic-based composites [5,6,7,8,9,10,11]. The calcite layers forming the columnar prisms elongated along the growth direction were investigated as well [12,13]. Prisms are usually considered as grains [14], but some studies revealed that they are built of some smaller crystallites [15,16]. Our previous investigations based on calculations of misorientation distribution functions [17] revealed the preferred orientation relationships between neighboring grains. These results suggest an ordering that is characterized by strong bonds [16]. The anisotropy of the microstructure and the occurrence of orientation dependencies in prismatic structures can be expected to influence the mechanical properties of mollusk shells.

The mechanical behavior of the calcite layer in the mollusk shells was investigated mainly through nanoindentation tests. Kunitake et al. [18] studied *Atrina rigida* mollusk shells, which also have a prismatic columnar layer in its structure using a Berkovich diamond indenter. The investigations revealed that the nanohardness might slightly vary depending on the plane exposed for the indentation. These results were confirmed by testing of the prisms at different azimuthal angles Φ interpreted as the angle between indented calcitic planes and indenter (~3.5 GPa and ~4.2 GPa for Φ = 0° and Φ = 60°, respectively). SEM microstructure observations showed that the number of cracks after nanoindentation test depends on the azimuthal angle too. The nanohardness of the individual calcite single crystal prism depends on its crystallographic orientation and may exceed 2.30 ± 0.14 GPa for {001} plane, while for calcite in mollusk shells, the value for the {001} plane is around 3.47 ± 0.21 GPa. Moreover, Perez-Huerta et al. [19] showed that there is a relationship between the orientation of a single calcite crystal, hardness, and Youngs modulus what suggests the presence of strong anisotropy of the shell’s mechanical behavior. However, the problem of anisotropy of mechanical properties of the prismatic columnar layer in the mollusk shells was not reported in the literature so far.

In the present work, the anisotropy of the mechanical properties of the prismatic columnar calcite layer, forming the outer part of *Pinctada margaritifera* mollusk shell was analyzed. The mechanical strength of the shell was evaluated through the static compression tests and with micro- and nanoindentation in two loading directions. The propagation of the cracks caused by micro-indentation was imaged using in-situ X-ray computed nano-tomography (nano-XCT). Additional microstructure observations of the sample and crack propagation after mechanical tests were performed using scanning electron microscopy (SEM).

## 2. Materials and Methods 

**Figure 1:**
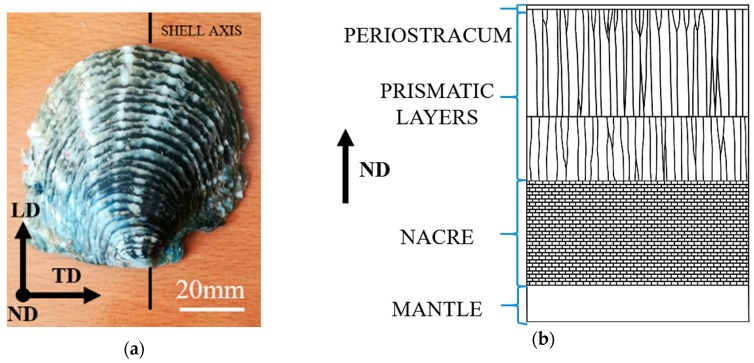
(**a**) Image of the outer surface of the *P. margaritifera* shell and definition of the directions where LD is the longitudinal direction parallel to shells axis, TD is transverse direction determined by growth lines on shells surface and ND is normal direction, indicating growth direction of the shell perpendicular to the plane defined by LD and TD and (**b**) schematic cross-section through the shell showing the layered structure.

*P. margaritifera* shells were obtained from Conchology Inc. company (Lapu-lapu, Cebu, Philippines). They were cut with the use of a diamond saw into small samples with dimensions of approximately 2 mm × 2 mm × 2 mm and polished to obtain flat surfaces. Throughout the paper, the principal directions of the samples are explained in Figure 1, i.e., longitudinal direction (LD) is parallel to shells axis, transverse direction (TD) which is perpendicular to shell axis and is determined by growth lines on shell surface and normal direction (ND) which is parallel to the shell surface. The samples were embedded in resin, ground with abrasive papers up to 7000 grit and polished with diamond suspensions with a grain size of 1 μm and 0.25 μm. 

The microstructure investigations of *P. margaritifera* shells were carried out using a Philips XL30 SEM (Eindhoven, Netherlands) operated at 10 kV acceleration voltage using BSE mode (BackScattered Electrons). The observations were performed before and after the mechanical tests in order to image the fractured surfaces. 

**Figure 2:**
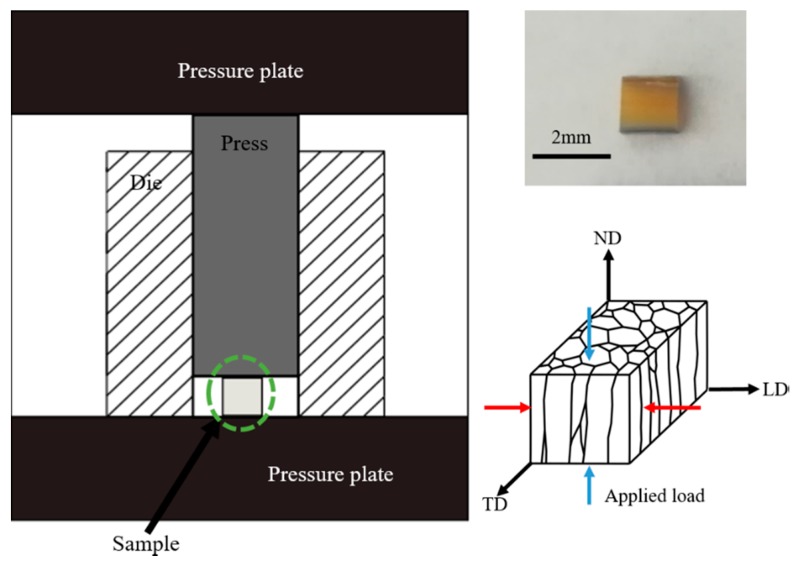
The scheme presenting the setup used for the determination of the strength of *P. margaritifera* shells and the image presenting the sample.

Mechanical properties of *P. margaritifera* shells were determined using uniaxial compression tests and nanoindentation measurements. In order to investigate only the outer, prismatic layer of the shell, the nacreous layer was removed using abrasive papers. The compression tests were performed in two loading directions, i.e., along with ND and LD using a Zwick/Roell INSTRON 6025 (Ulm, Germany) testing machine and a hardened steel die at room temperature (~22 °C) with a constant strain rate of 10^−3^ s^−1^. For each investigated shell direction, five samples were tested. A scheme presenting the setup used for the determination of the strength of the shells is presented in Figure 2. In order to obtain the values of the hardness and the Young modulus of the material, the nanohardness measurements were carried out with the use of Bruker Hysitron TI950 setup (Minneapolis, MN, USA). Indentations were carried out at a maximum load of 10 mN with Berkovich tip resulting in average penetration depths of around 371 nm and 238 nm for loading applied along with ND and LD, respectively. The loading rate was 500 µN/s. All the indents (100 for each sample) consisted of 20 s load, 10 s hold, and 20 s unload stages. The load applied during nanoindentation was too small and did not allow observing how the cracks propagate inside the material, the micro-indentation was performed. The measurements were carried out with the use of a CSM Instrument tester (Peseux, Switzerland) equipped with Vickers indenter under a load of 1N (HV0.1). 

Nano-XCT studies were carried out in order to obtain a 3D visualization of the microstructure of calcitic prisms forming the outer layer of the shell and of cracks that appeared after the indentation process with a resolution of about 100 nm. The combination of nano-XCT studies with in-situ micromechanical experiments merges the advantage of nondestructive high-resolution 3D imaging of the microstructure of materials with the observation of phenomena like crack initiation, crack propagation, and delamination during mechanical loading. For these studies, a laboratory transmission X-ray microscope Xradia Nano-XCT-100 (Concord, California) operated at a photon energy of 8.0 keV (Cu-Kα radiation) [20,21,22] was used during the micro-indentation test to visualize the crack propagation. A detailed experimental setup was described elsewhere by Zgłobicka et al. [23]. For these experiments, small rectangular specimens with a cross-section of approximately 50 µm × 50 µm and a length of 200 µm were prepared using diamond disc sawing and subsequent polishing. The specimens were fixed to the rod-shaped sample holders according to assumed orientation with the use of an optical microscope. Before and after the indentation test, complete tomography data were acquired, and a 3D reconstruction was done. It consisted of 401 images collected during 180° rotation of the sample with exposure times of 140 s for each image. The full tomography before the indentation test was done in order to check the integrity of the sample and to define the setting of the indenter for the in-situ indentation process. The mechanical loading of the shell was performed within the nano-XCT system using a customized micro-mechanical testing system which was designed for in-situ micro-indentation experiments in such a way that it fits into the limited space of the nano-XCT tool in particular and that the indenter tip and the sample fits into the field of view of the X-ray microscope. The system has been described by Zschech et al. [21]. The indentation tests in the XCT were performed, the same as compression tests, along two directions, i.e., ND and LD, using a cube corner tip. In order to track the crack propagation during the in-situ XCT indentation test and to prevent the destruction of the sample, the reference images were acquired with an exposure time of 120 s after each loading step of approximately 10 mN. The test was stopped immediately as soon as large visible cracks occurred. The tomography data acquired after the indentation test allowed the 3D presentation of the crack pattern. The 3D reconstructions were generated with Fiji software [24] and proprietary software installed in the instrument (Xradia XMReconstructor, Pleasanton, CA, USA). 

## 3. Results

### 3.1. SEM Microstructure Observations of As-Received P. margaritifera Shells

**Figure 3:**
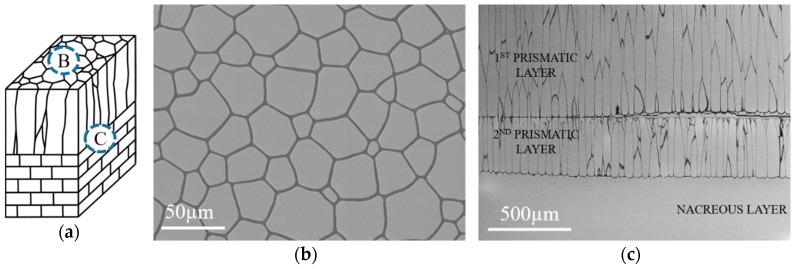
Schematic cross-section of *P. margaritifera* mollusk shell with marked areas of SEM observations (**a**) and corresponding SEM/BSE microstructure images acquired from the section perpendicular to ND (**b**) and LD (**c**).

Figure 3a presents schematic cross-section of investigated shell. The SEM/BSE microstructure observations of the top view of the polished prismatic layer (i.e., on the section perpendicular to ND) of the as-received *P. margaritifera* shell revealed that it is built of simple, almost hexagonal polygons with different size ranging from ~10 µm to ~80 µm. They are separated by organic sheets visible in the images in the form of thin dark layers having few micrometers width (Figure 3b). The observations carried out in the cross-section parallel to ND showed that the investigated mollusk shell is composed of two outer columnar prismatic layers with a thickness of ~920 µm and ~350 µm for the first and the second layer, respectively, and the inner layer of nacre (Figure 3c). The size of the prisms changes dramatically. It is caused by the fact that at first small grains start to form, and at later stages of the shells growth, they enlarge. That is why on the cross-section longitudinal to shell growth (LD), one may observe very small grains at the top of the shell and very big prisms, which sometimes occur across the entire width of the layer. Thus, the average widths of these columns were measured, and their values were 41 µm and 34 µm for the 1st and 2nd prismatic layer, respectively. 

### 3.2. Uniaxial Static Compression Testing of P. margaritifera Shells

**Figure 4:**
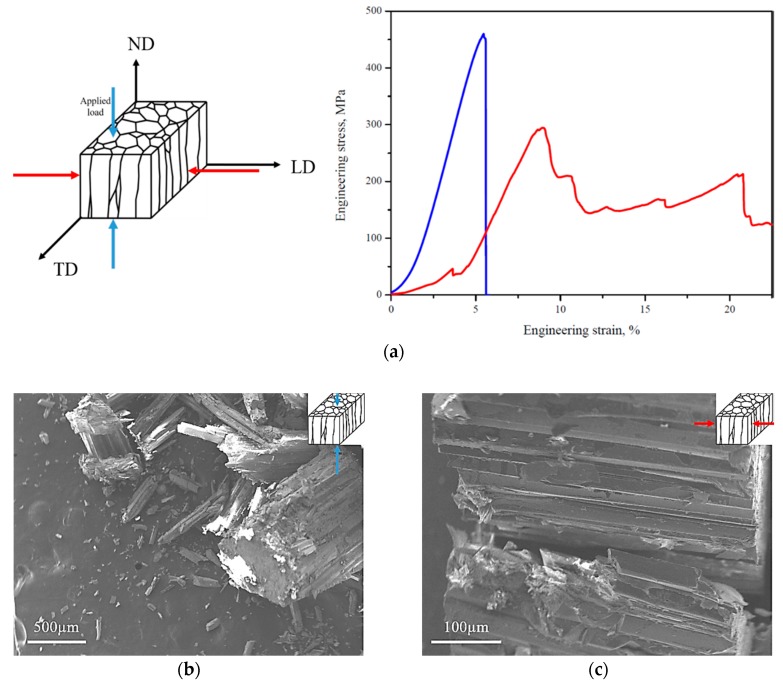
Representative stress-strain curves obtained in the static compression test for *P. margaritifera* performed along ND and LD (**a**) and SEM/SE images of the samples after static compression test for loads applied along ND (**b**) and LD (**c**).

The representative stress-strain curves obtained during the uniaxial static compression testing of the outer layer of *P. margaritifera* shell vary significantly depending on the direction of the applied load, as shown in Figure 4a. If the compression of the sample was performed along ND, a brittle fracture, typical for ceramic materials, was observed, i.e., the samples ruptured without any plastic deformation. In this case, the material presents a high compressive strength, which was measured to be 460 ± 100 MPa. On the other side, if the load was applied along LD, the serrated stress-strain curve, typical for ceramic composite materials, was obtained. The average value of compressive strength of 220 ± 90 MPa was measured. The spread of compression strength values is a result of the biological origin of samples. In such samples, the content of the organic phase may change as well as the thickness of the layers forming of the material. However, the most important thing was that for all the samples loaded along the same direction, the curves had a similar shape revealing some tendency of the material to behave, in particular way, when the load was applied. Therefore, only the representative ones were presented in Figure 4a.

The SEM/SE images of the *Pinctada margaritifera* shell after the static compression test performed along ND and LD show that the cracks run predominantly along the prisms (Figure 4b,c). In the first case, the samples were mostly divided into the stacks of prisms, but the crushed individual prisms may also be observed. If the load was applied along LD, the prisms placed on themselves are sliding on organic sheets. Most of the prisms retained their original shape, and the layers of the organic material were clearly delaminated (Figure 4c). It suggests that the material ruptured via the organic parts. 

### 3.3. SEM Observations of P. margaritifera Shells After Micro-Indentation Test

**Figure 5:**
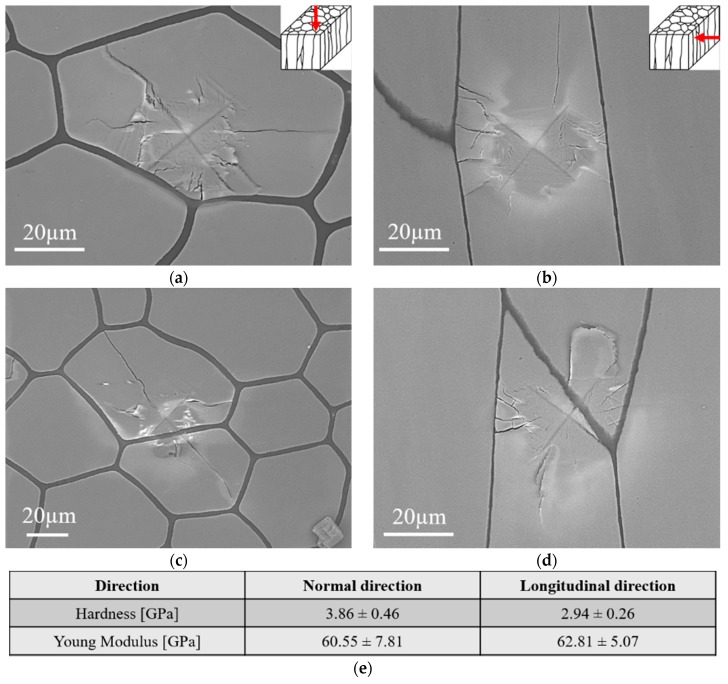
SEM/BSE microstructure images presenting the indents acquired from the section perpendicular to ND (**a**,**c**) and LD (**b**,**d**) and the measured with nanoindentation values of hardness and Young modulus for each direction (**e**).

The SEM/BSE microstructure observations of *P. margaritifera* shells after the micro-indentation test revealed the presence of the cracks propagating in the prisms (Figure 5). It is clearly seen that these cracks are not only formed near to the edges of the tip but in all sides of the indent and that they propagate across the individual calcite prism to the organic part, where they are stopped. This shows that the layer of soft organic part plays the role of an obstacle for crack propagation, preventing the catastrophic failure of the mollusk shell during the loading. When the load was applied along LD, the cracks also propagated in the individual prism until the organic phase is reached. However, some cracks propagate parallel to the organic layer (Figure 5b,d). The results of the nanoindentation test revealed that the hardness value strictly depends on the direction of investigations reaching ~4 GPa for ND and ~3 GPa for LD, respectively (Figure 5e). When the indenter is pressed into the organic part, the cracks propagate through two neighboring prisms until they reach the organic part (Figure 5c,d).

### 3.4. In-Situ XCT Imaging during Micro-Indentation of P. margaritifera Shells

**Figure 6:**
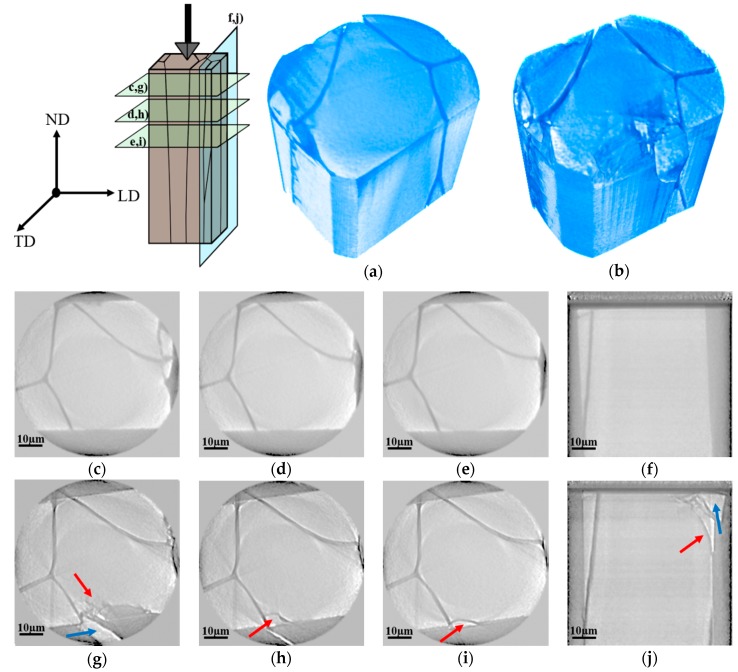
Scheme of in-situ X-Ray Computed Tomography indentation of *P. margaritifera* shells along ND, 3D reconstruction of the sample before (**a**) and after (**b**) in-situ indentation test; horizontal and vertical virtual cross-sections of the shell acquired before (**c**–**f**) and after (**g**–**j**) the indentation test (red arrows point to the cracks, while blue arrows show part of the material damaged during the test).

**Figure 7:**
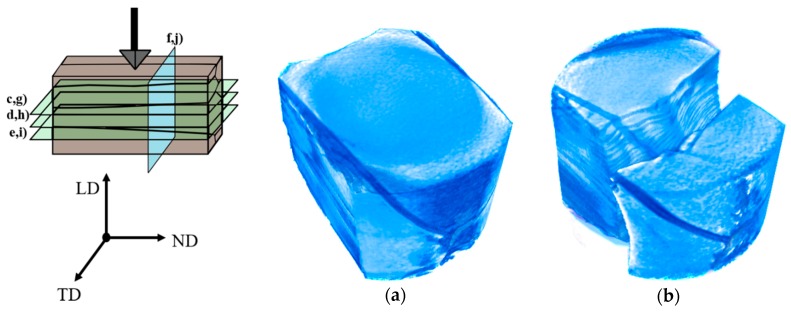

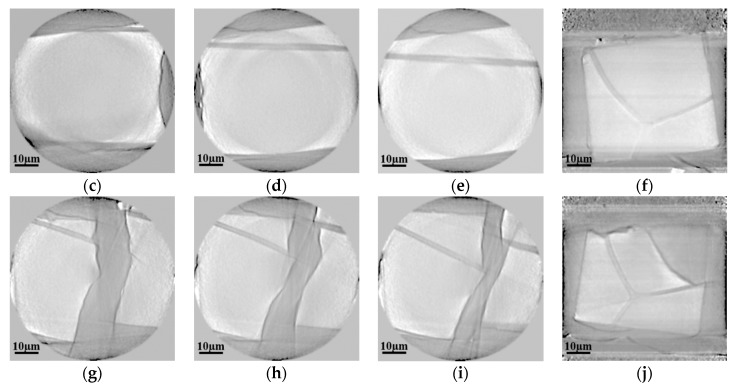
Scheme of in-situ X-Ray Computed Tomography indentation of *P. margaritifera* shells along LD, 3D reconstruction of the sample before (**a**) and after (**b**) in-situ indentation test, horizontal and vertical virtual cross-sections of the shell and 3D reconstruction acquired before (**c**–**f**) and after (**g**–**j**) the indentation test.

The X-ray images and 3D reconstruction of *P. margaritifera* shells acquired before and after the indentation test performed on a section perpendicular to ND and LD are presented in Figure 6 and Figure 7, respectively. They allowed the confirmation of the tendency of cracks to propagate deep into the calcite prisms and to stop in the organic part. For the sample investigated on the section perpendicular to ND (Figure 6) two different crack mechanisms were observed. In the first case, the cracks spread on the surface and propagate to the ductile organic part where they are stopped. In the second case, when the prism is not limited by the organic part, the cracks lead to the catastrophic failure and removal of the part of the material (Figure 6b). The maximum crack propagation depth was measured on the section perpendicular to ND was ~24 µm, taking into account that the indenter penetration depth was ~8 µm. In the case of the sample indented on the section perpendicular to LD (Figure 7), critical damage of the material may be observed. Thus, one may conclude that bare calcitic prisms are very brittle and weak material, and they behave similarly to the mineral calcite. What is more, in this direction, very small amount of organic part is observed, which, in this case, is unable to stop cracks propagation. The indentation test revealed that the crack would propagate through neighboring prisms and stop on the further located organic part. Critical damage to the material may be observed due to the lack of the organic layer.

## 4. Discussion

The outer layer of the *P. margaritifera* shell consists of a simple, mutually parallel calcite prisms, arranged with their crystallographic c-axes perpendicular to the shell surface. These so-called “prisms” are polygonal (hexagonal on average) in cross-section. This microstructure is expected to have a significant influence on the mechanical properties of the shells and particularly on the way of crack propagation during mechanical loading.

The expected strong anisotropy of the mechanical behavior was experimentally proved in the uniaxial static compression test as the shape of the stress-strain curves, as well as the ultimate compressive strength, definitely varied depending on the loading direction. A brittle cracking was observed if the compression was performed along ND. The samples ruptured without any plastic deformation what is characteristic of ceramic material. When the load was applied along LD after reaching the ultimate strength, stresses oscillate around lower values. Such stress-strain curves are typical for natural ceramic-based composites. Similar behavior of the stress-strain curve was observed in different natural and synthetic composites as bone, wood, or nacre-like materials [25]. This observation is consistent with delamination of the prisms and gliding of the hard, ceramic prisms along the soft organic part, which was confirmed through the microstructure observations. 

The propagation of cracks formed in the calcite prisms during the indentation test was documented by SEM microstructure observations (2D imaging) and 3D reconstruction of nano-XCT data. Most of the cracks were extinguished in the soft and ductile organic part. These experimental results confirm the significant role of the organic part for the crack evolution in natural composites such as shells [26,27]. The thin organic layers which connect the brittle mineral units increase the fracture resistance of a whole hierarchically structured materials system [28]. However, some cracks may propagate along an individual prism leading to a catastrophic failure of the material. The observed different cracking behavior depending on the studied cross-section is strictly connected with the microstructure of the outer layer of the shell, which generates the anisotropy of mechanical properties. 

Observed within this paper anisotropy of mechanical strength and cracking behavior of the outer layer of *P. margaritifera* shell was “developed” in evolutionary time. The mollusk has to be protected mainly along ND as the predator will attack along this direction and, therefore, the ultimate compressive strength measured along ND needs to be as high as possible. We consider that the microarchitecture of the *P. margaritifera* shell provided by Nature during evolution may state a basis for the design and manufacturing of a new class of materials characterized by desirable mechanical and functional properties. What is more, such a simple composite structure with the ability to stop cracks may be useful in systems where microcracks lead to critical destruction of materials. The constructions may be protected from catastrophic failure by a specific arrangement of the hard-phase columns separated from each other by thin layers of the soft matrix, which constitute the paths for cracks. 

## 5. Conclusions

In the present study, the anisotropy of mechanical properties of the outer columnar prismatic calcite layer of *P. margaritifera* mollusk shell was studied. The microstructure observations performed after static compression and indentation tests backed by 3D reconstruction of XCT images after indentation test allowed to conclude that:

- Brittle cracking of the shell, typical for ceramic materials, was proven for the load applied along the ND complex. The serrated stress-strain curve was documented when a compression was performed along LD, which is an evidence of the sliding of calcite prisms on the layers of the soft organic part
- The shell is characterized by significantly higher ultimate compressive strength and hardness along ND than LD
- Cracks formed in a single calcite prism under a load of indenter revealed a tendency to propagate along the prism and to stop at the ductile organic part acting as an obstacle. If the calcite prism was not limited by the organic part layer, the crack path would proceed until the edge of the sample was reached and would lead to its catastrophic failure.

The above results confirm the strong anisotropy of mechanical properties of the studied structure. Prisms act as reinforcement arranged along the expected direction of load. The organic part contained in them contributes to a significant improvement in the compressive strength of the material by trapping cracks and deflecting their propagation paths. Further research is needed to fully identify the energy dissipation mechanisms included in the prismatic structure of the protective armor as well as to indicate the crystallographic planes on which the cracks propagate. Using them in synthetic composites based on reinforcement with a much higher strength than in the case of calcite will allow designing materials with unique mechanical properties.
